# Exploring the role of resilience in selective prevention intervention for adolescents at risk of depression and anxiety in Nepal: findings from a pilot cluster randomized controlled trial

**DOI:** 10.1186/s13034-025-00964-8

**Published:** 2025-09-29

**Authors:** Rakesh Singh, Parinati Khanal, Wietse A. Tol, Philip Jefferies, Mark J. D. Jordans, Crick Lund

**Affiliations:** 1 Centre for Global Mental Health, Health Service and Population Research Department, Institute of Psychiatry, Psychology and Neuroscience, King’s College London, 18 De Crespigny Park, London, SE5 8AF UK; 2 Research Department, Transcultural Psychosocial Organization Nepal, Baluwatar, Kathmandu Nepal; 3 Department of Public Health, Section for Global Health, University of Copenhagen, Copenhagen, Denmark; 4 VU University Amsterdam, Amsterdam, the Netherlands; 5 Atlantic Technological University, Castlebar, Mayo, Ireland; 6 Department of Psychiatry and Mental Health, Alan J Flisher Centre for Public Mental Health, University of Cape Town, Cape Town, South Africa

**Keywords:** Anxiety, Depression, Psychological resilience, Mediation, Prevention, Adolescent interventions

## Abstract

**Background:**

Understanding the mechanisms of change has recently been emphasized as crucial for advancing research on preventive interventions. This study, embedded within a pilot trial, aimed to explore the mediating effects of resilience for three preventive interventions focusing on reducing the risk of adolescent depression and anxiety in Nepal. We hypothesized that the self-regulation, economic, and combined interventions would differentially increase internal and external resilience, which in turn would reduce adolescent depressive and anxiety symptoms among adolescents in Nepal, supporting to development of a testable, replicable mediation model of prevention intervention pathways.

**Methods:**

Data were collected as part of a feasibility cluster-randomized controlled trial. A total of 229 adolescents aged 13–15, identified as living in poverty and at risk of depression or anxiety, were cluster-randomized by school into three intervention arms (self-regulation, economic, combined) and a control group. Assessments for depression and anxiety symptoms, and external and internal resilience were performed at three timepoints, with a six-month interval between each. Twenty school-based group intervention sessions were conducted weekly post-baseline. Linear mixed modeling explored changes in resilience within groups. Exploratory mediation analyses were performed to examine the association between interventions (as predictors), resilience at 6 months (as mediator), and symptoms of depression and anxiety at 12 months as outcomes, through two separate parallel mediation models.

**Results:**

While no significant sensitivity to change effects for resilience was found, hypothesized directional time-related improvements were observed in external resilience for males in self-regulation and economic arms, and for females in the self-regulation arm; internal resilience showed positive trends for males in economic and combined arms, and for both males and females in the self-regulation arm. No significant effects of the interventions through resilience on mental health outcomes were detected, though internal resilience at 6 months predicted lower anxiety at 12 months, and for males in all intervention arms, higher internal resilience was significantly associated with lower depression.

**Conclusions:**

The findings warrant the testable conceptual mediation model with resilience as a mechanism in larger, fully powered prevention trials for adolescents.

*Trial registration:* ISRCTN14601588 https//doi.org/10.1186/ISRCTN14601588.

## Introduction

Depression and anxiety are the leading causes of disease burden among adolescents worldwide [1], posing risks to healthy development and long-term well-being by contributing to later mental health problems, physical health issues, challenges in education and employment, and lower life satisfaction in adulthood [2, 3]. Globally, an estimated 9.9% of adolescents experience anxiety, and 4.9% suffer from depression [1]. Typically, depression and anxiety have their onset during adolescence, with most conditions often occurring around the age of 14 [4–7]. About 80% of the global burden of depression and anxiety is concentrated in low- and middle-income countries (LMICs) [8], where 90% of the world’s adolescent population resides [9].

Mental health risks in adolescents in LMICs are intensified by the complex interplay of factors, including poverty, limited mental health infrastructure [10], and stigma surrounding mental health [11]. With a high rate of mental health issues but limited mental health services, evidence-based interventions to promote resilience and to prevent risk factors of anxiety and depression among adolescents in low-resource settings have been highlighted as an urgent priority [12]. Selective prevention strategies targeting adolescents with risk factors for depression and anxiety can be effective in LMICs [13, 14]. Studies in high-income countries (HICs) have demonstrated the effectiveness of school-based interventions in reducing adolescent depression and anxiety symptoms [15–17]. However, the evidence on the effectiveness of preventive interventions for depression and anxiety among adolescents in LMICs remains limited [18, 19], particularly across diverse contexts, which may constrain the applicability of interventions due to cultural, economic, and contextual differences [20].

A research project entitled *improving adolescent mental health by reducing the impact of poverty* (ALIVE) [21] aimed to develop and pilot test a selective prevention intervention that targets both psychological and economic determinants to prevent depression and anxiety in adolescents living in poverty in low-resource contexts. The ALIVE pilot trial consists of three types of interventions: a self-regulation-only intervention, an economic-only intervention, and a combined intervention, which includes both self-regulation and economic interventions. The ALIVE selective prevention interventions (self-regulation, economic, and combined) were developed based on a conceptual framework linking poverty, self-regulation, and depression and anxiety among adolescents. The overarching premise of this framework is that interventions that combine psychosocial components improving self-regulation with poverty reduction strategies have the potential to prevent depression and anxiety among adolescents [21]. The active ingredients of the interventions were based on existing literature on interventions that strengthen self-regulation [22–26] and reduce the impact of poverty [27–30]. Furthermore, reducing the impact of poverty has been demonstrated to strengthen self-regulation [21]. The findings from the ALIVE formative work on self-regulation strategies used by adolescents were subsequently integrated into the intervention curricula [31].

Some studies have shown that self-regulation interventions [32] and economic interventions (cash transfers only) [33–35] can reduce depression and anxiety among adolescents in LMICs; however, some have argued that solely providing cash transfers may not be sufficient to promote mental health outcomes [36]. Previous studies have recommended incorporating additional components such as financial education, life skills training, and use of a safe space to the cash alone intervention (i.e., economic intervention with cash plus program) to overcome stressors due to poverty and making it more effective in reducing depression and anxiety [37, 38]. Given the complex nature of poverty [39, 40] and the diverse contextual stressors affecting adolescents in LMICs, it is essential to further explore the effects of the cash-plus economic intervention, self-regulation intervention, and their combination—especially in light of the mixed findings in the literature and the context-dependent nature of these outcomes.

A few recent studies have found that combined interventions (employing cognitive-behavioral techniques and enhancing social capital, education, and literacy levels) were effective in reducing depression and anxiety in adolescents in low-resource settings [41, 42]. Despite evidence supporting the effectiveness of preventive interventions, both self-regulation intervention and economic intervention as ‘stand-alone’ or ‘in combination’, in reducing depressive and anxiety symptoms [42–46] in adolescents, there is limited understanding of the mechanisms through which these interventions work [47].

Understanding how interventions bring about change—by exploring the key factors they target and influence—is important for improving the intervention’s design and effectiveness. Exploring the mechanisms through which interventions operate can inform how the core components might be tailored or optimized to enhance their impact on outcomes [48]. Examining the mediational effect in the prevention interventional research is very important even in the absence of an intervention effect; mediation analysis can reveal underlying mechanisms of change, provide critical insights into why interventions succeed or fail, and extract valuable information that enhances understanding of complex behavior change processes, making it an essential tool for evaluating and refining intervention models [49].

Research has focused on processes of resilience that can explain how adolescents exposed to adversity retain and regain well-being. Modern resilience theory emphasizes that resilience evolves from both internal and external protective factors [50], shaped by ongoing interactions between individuals and their environments [51, 52]. Resilience plays a protective role in reducing depression and anxiety [53], with adolescents having higher resilience being less likely to experience depression and anxiety [54–57]. Internal protective factors include optimism, problem-solving skills, a sense of purpose, and perseverance [57–61], and external protective factors involve access to supportive family relationships, peers, schools, and broader social networks [57, 62]. Ecological resilience [63] considers how individual functioning is shaped by the dynamic interplay of risk, protective, and promotive factors operating at multiple levels, including the individual, family, peer group, and community [64–66]. Interventions based on this model aim to strengthen coping skills, hope, and social support—factors which enhance adaptive development in adverse environments [67, 68]. Protective factors are not just outcomes but active components of the resilience process, and as such, they have been identified as important targets and potential mediators within interventions aimed at reducing symptoms of depression and anxiety. For example, optimism, positive emotions, and self-compassion, as processes, were improved by the positive psychology interventions and mediated the interventions in reducing depressive and anxiety symptoms [69]. However, there remains a limited understanding of how such interventions produce change, with many lacking an articulated model describing the mechanisms involved. This gap presents a critical opportunity to contribute to intervention science by advancing conceptual models that clarify how and why interventions work.

In a recent cross-sectional study conducted as a part of ALIVE, we found that external resilience had a mediating effect on the relationship between poverty and depressive and anxiety symptoms among adolescents in Nepal [70]; however, it lacked the temporality of the mediation effect, making it difficult to establish whether the mediators preceded and influenced the outcome over time. Also, its potential mediating role has not been explored adequately within the context of preventive interventions targeting adolescent depression and anxiety, in Nepal, one of the poorest countries in South Asia facing challenges such as high poverty rates [71], limited mental health services, a shortage of trained mental health professionals, stigma preventing individuals from seeking help for mental health issues [72, 73], and a lack of preventive mental health services for adolescents [74]. Understanding whether economic-only intervention (cash plus financial education program), self-regulation-only intervention, or a combination of both interventions (economic plus self-regulation) function as pivotal protective factors to enhance resilience and improve adolescent mental health outcomes can inform the development of effective, scalable, and cost-effective prevention strategies. This is particularly critical given that adolescence is a period of significant personal and social transformation, making resilience a key protective factor during this developmental stage [62].

Embedded within the broader ALIVE conceptual framework for modular selective interventions linking poverty, self-regulation, and adolescent depression and anxiety [21], in this study, we hypothesized that self-regulation and resilience are positively correlated, and resilience is negatively correlated with depressive and anxiety symptoms in adolescents. The current study aimed to (i) explore the sensitivity to change in resilience (internal and external) as exploratory outcomes over time (baseline to 6 months and 12 months) within ALIVE intervention arms (self-regulation, economic, combined, control)and (ii) explore the possible fit of the mediation model to assess the mediational role of resilience (both internal and external) at 6 months in the relationship between intervention arm allocation (with control as the reference) at baseline and depressive and anxiety symptoms at 12-month follow-up. We explored whether the ALIVE interventions may increase resilience (internal and external), which in turn could contribute to reducing adolescent depressive and anxiety symptoms in Nepal. The study, comprising three types of interventions targeting economic resources (cash plus financial literacy, negotiation skills, and information on returns to education), self-regulation skills, and their combination, aligns with both the Developmental Systems Framework of resilience [75] and the adaptation-based approach to resilience [76]. From a systems perspective, resilience emerges from dynamic interactions across multiple, interconnected levels, including the individual, family, peers, schools, and broader community, with changes in one system cascading to influence others over time. Simultaneously, the adaptation-based approach emphasizes that adolescents growing up under harsh or adverse conditions, such as poverty, develop cognitive, social, and behavioral skills through trade-offs in resource allocation—prioritizing context-specific adaptive benefits that may enhance immediate coping over long-term gains. Integrating these perspectives, this study aims to evaluate how strengthening adolescents’ internal capacities and enhancing external protective factors can leverage existing stress-adapted strengths to promote adaptive development and prevent depression and anxiety in the context of Nepal’s collectivist socio-cultural environment. This combined theoretical lens recognizes resilience as both distributed across interacting systems and adaptively specialized, providing a rationale for multi-component interventions that work with, rather than against, the developmental adaptations of adolescents in adverse contexts such as poverty. The goal of the study was to assess whether there were trends in the expected direction with regard to differences within-groups from baseline to follow-up periods, and to develop a testable, replicable, interdisciplinary mediation model for a fully-powered ALIVE trial, with resilience as a key mechanism in the prevention intervention.

## Methods

### Study design

This study was nested within the ALIVE parallel 4-arm pilot cluster randomized controlled trial (cRCT). The study arms included a self-regulation intervention, an economic intervention, a combined (self-regulation + economic) intervention, and a control group. The trial was registered on 19 May 2024 (ISRCTN 14601588). In Nepal, the current study was conducted among adolescents aged 13–15 years enrolled in public secondary schools of Budhanilkantha municipality and its adjacent wards of Kathmandu metropolitan city. These areas were selected for their high vulnerability to poverty, aligning with ALIVE’s focus on urban poverty, and feasibility for school-based recruitment. Of the 21 public secondary schools in the study area, 11 were eligible based on the inclusion criteria for the public schools requiring at least 100 adolescent students across grades 6–8 and proximity between two schools (> 1 km). Eight eligible schools were then randomly selected and considered adequate to meet the required sample size for the ALIVE project [21, 70]. These eight public secondary schools from Budhanilkantha municipality and Kathmandu metropolitan city were then randomized equally (1:1:1:1) (schools as clusters with two schools per arm) across the four arms using computer-generated pseudo-randomization. The randomization was conducted before the baseline data collection.

### Study participants

**Fig. 1:**
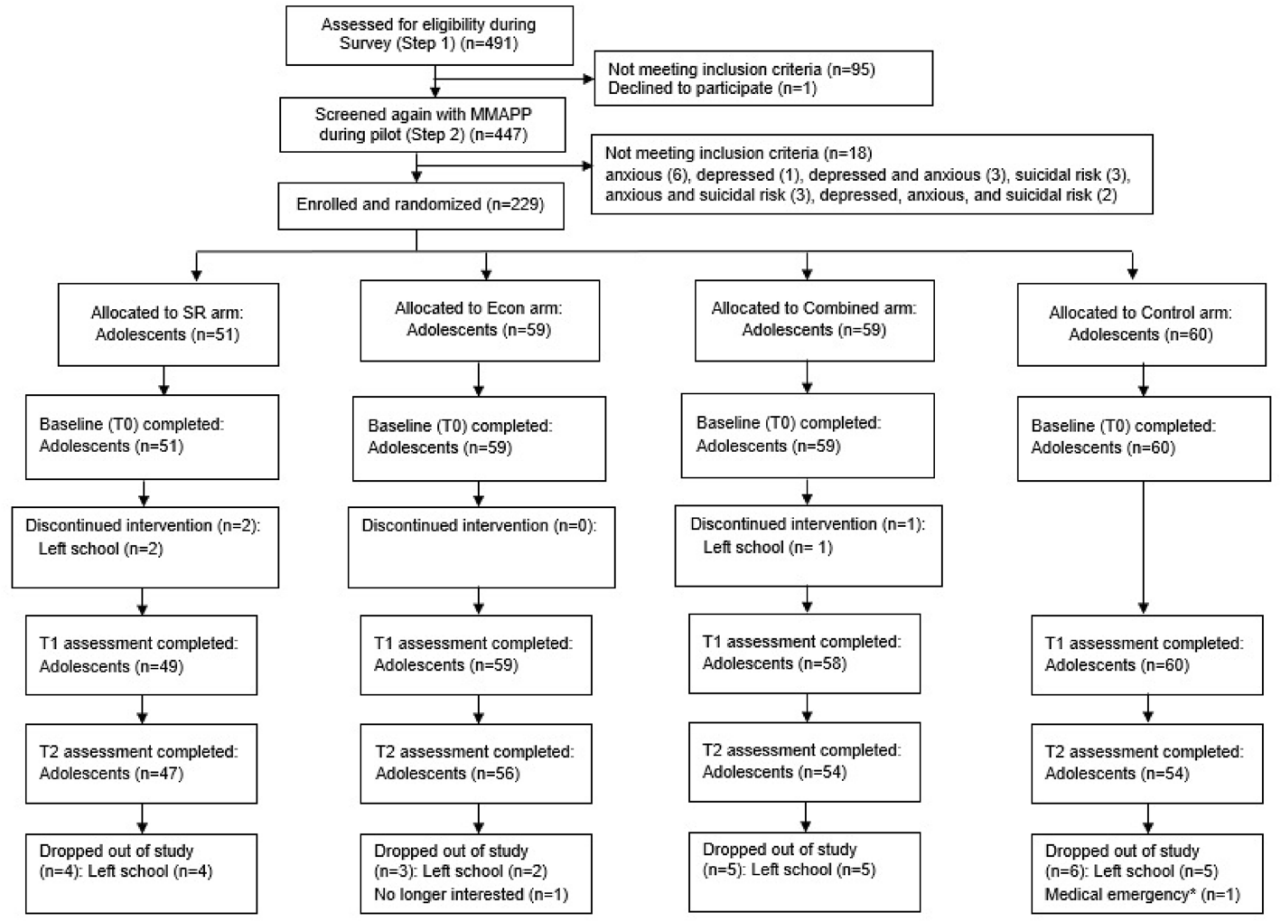
CONSORT flow chart

The participants’ inclusion criteria for their selection included – (1) adolescents attending public secondary schools with a positive screen on the 8-item poverty screening instrument, (2) aged between 13 and 15 years at the time of first phase of recruitment, (3) fluent in the Nepali language, and (4) not identified as having depression or anxiety, defined as having scores below 15 on the Patient Health Questionnaire-Adolescent (PHQ-A) for depression and below 10 on the Generalized Anxiety Disorder (GAD-7) instrument for anxiety. Adolescents aged 13–15 years at the time of recruitment were considered for inclusion, as depression and anxiety typically have their onset during adolescence [4–7]. This age range was selected to align with the preventive focus of the intervention. Limiting the study participants to this age range may introduce age-related selection bias, as early- or late-onset cases (those below 13 or above 15 years) are excluded, which could potentially affect generalizability. However, because participants were followed up longitudinally after interventions (12 months post recruitment), the study was able to track the emergence of depressive or anxiety symptoms beyond age 15, thereby partially addressing the exclusion of late-onset cases. The recruitment of adolescents within clusters (schools) took place in two phases. In the first phase, 491 adolescents were enrolled from eight schools, after selecting classes (from grades 6–8) representing the study age range of 13–15 years; this was performed as a part of the cross-sectional study to examine the associations among depression, anxiety, resilience, and economic indicators (described in detailed somewhere else [77]), . In the second phase, we randomly selected approximately 30 participants per school who were at risk of depression or anxiety, defined by those not having depression (PHQ-A score < 15) or anxiety (GAD-7 score < 10) among those who were enrolled in the first phase cross-sectional survey. In the second phase of recruitment, adolescents at high suicide risk, defined as reporting current suicidal ideation with intent or plans over the past month, or reporting a suicidal attempt in the past 3 months, were excluded. A total of 229 adolescent participants were enrolled at baseline (T0), with 51–60 participants per arm (i.e., 20–30 adolescents per school). Participants were free to withdraw from the intervention and/or study at any time without any consequences. A total of 3 participants were lost to follow-up at the 6-month follow-up (T1), and 18 participants were lost at the 12-month follow-up (T2). The detailed recruitment process and loss to follow-up during the intervention and after every assessment (T0, T1, and T2) are presented in the CONSORT flow chart (Fig. 1). Participants in the intervention arms were classified as completers if they attended ≥ 70% of the 20 sessions. Completion rates for interventions were 58/59 (98%) in the economic arm, 46/51 (90%) in the self-regulation arm, and 57/59 (97%) in the combined arm. At T2, the number of dropouts was 3 in the economic arm, 4 in the self-regulation arm, 5 in the combined arm, and 6 in the control arm (Fig. 1). Among these, all 3 dropouts in the economic arm were completers, as were 4 of 5 in the combined arm and 2 of 4 in the self-regulation arm. Although most of the dropouts from the intervention arms were classified as completers, this is unlikely to affect the study outcomes, given the high adherence and consistently high completion rates observed across all intervention arms. This study is embedded within the larger Pilot study in Nepal; the latter will be reported separately following CONSORT guidelines.

### Measures

Poverty screening was conducted using an eight-item screening tool adapted from the Multidimensional Poverty Index for the ALIVE study to capture adolescents’ experiences of deprivation in the local context [21]. The tool drew on indicators commonly used in national surveys and covered domains including household income (regularity of income, income relative to the national poverty line, and dependency ratio), educational attainment of the household head and other members, and household access to basic facilities (private toilet and kitchen) and key physical assets. Each item was weighted (1/9 for the three income- and dependency-related indicators, 1/6 for the two education-related indicators, and 1/9 for the three sanitation and asset indicators) to calculate a composite deprivation score. Adolescents with scores ≥ 0.33 were classified as living in poverty. Although the tool has not undergone formal psychometric testing, it was developed through expert consultation and piloted during the ALIVE tool adaptation process to ensure contextual appropriateness and comprehensibility. For example, preliminary validation in Nepal showed that among 67 participants included in the pretesting, 62 screened positive for poverty, and subsequent qualitative assessments supported the tool’s contextual and construct relevance for identifying individuals living in poverty.

For assessing depressive and anxiety symptoms, the validated tools, PHQ-A and GAD-7, for adolescents in the context of Nepal [78], as a part of the measurement of mental health among adolescents and young people at the population level (MMAPP) tool [79] were used to generate equivalency scores for the PHQ-A and GAD-7 [78], both as continuous scores. Higher scores on the PHQ-A and GAD-7 indicated greater symptoms of depression and anxiety, respectively. The 17-item Child and Youth Resilience Measure-Revised (CYRMR) [80] was used to assess external resilience, with preliminary validation in Nepal [77]. The 10-item Rugged Resilience Measure (RRM) [81] was used to measure internal resilience, with preliminary validation in Nepal [77]. Both external and internal resilience were computed as continuous scores; higher scores indicated higher resilience. For self-regulation, a reduced version of the difficulties in emotion regulation scale – short form was used [82], with preliminary validation in Nepal as a part of the ALIVE tool adaptation study [21]; higher scores indicated higher issues in self-regulation.

### Procedures

The pilot trial received ethics approval on 18 March 2024 from King’s College London’s Health Faculty Research Ethics Subcommittee (reference HR/DP-23/24-40543) and the Ethical Review Board of the Nepal Health Research Council (reference number 1938, protocol registration number 167_2024). The permissions were also obtained from the education units of the Budhanilkantha municipality, and Kathmandu metropolitan city, and the school principals of all eight schools. Written informed consent and assent were obtained from adolescent participants and their caregivers before their enrollment in the study. The participants’ enrollment in the pilot trial and their baseline survey (T0) were conducted from April 21 to May 4, 2024. The intervention implementation occurred between 13 May and 4 September 2024. The post-intervention survey (T1) was done in September 2024, and the 12-month follow-up (T2) was from April 28 to May 17, 2025. The face-to-face interviews from T0-T2 were conducted within the school premises by trained research assistants using Open Data Kit (ODK) via Android tablets.

### Interventions

A total of 20 group-based sessions spread over the period from 13 May and 4 September 2024 were implemented across the three intervention arms. There were two groups in each of the clusters across all three intervention arms, and each group consisted of 10–15 adolescent participants. A session averaged 90 min for self-regulation and economic intervention sessions, and around 2 h and 15 min for combined intervention sessions. Session timing varied across the study arms for two main reasons: first, it depended on the time slots that schools within each arm made available for us to implement the sessions; and second, we intentionally tested different delivery timings (e.g., during school hours, before school hours, or on holidays/weekends) to assess feasibility and inform planning for a future trial. In the combined arm, most sessions occurred during school hours (16/20, 80%), with the remainder held during holidays (4/20, 20%). In the self-regulation arm, sessions were primarily before school hours (13/20, 65%), with some during holidays (6/20, 30%) and a few during school hours (1/20, 5%). In the economic arm, most sessions were during school hours (14/20, 70%), with the remaining sessions during holidays (6/20, 30%). All sessions were conducted in schools, with each session implemented by two facilitators per arm, totaling 12 facilitators across the three arms. A cascading model for capacity building was used to train facilitators in the three interventions. Intervention developers served as master trainers and conducted a five-day Training-of-Trainers (ToT) workshop in Cape Town in February 2024 for two trainers per intervention. Trainer selection criteria were aligned with intervention content and took into account the available staff cadre and resources in the country—for example, a postgraduate diploma or bachelor’s degree in psychology or a related field with experience in psychosocial trainings (for self-regulation component), or a bachelor of arts in business studies or a related field with understanding of basic financial management skills such as budgeting and experience in conducting training on financial, cooperative, or microfinance management (for economic component). The two trainers per intervention per country subsequently trained four facilitators per intervention during a six-day training in Nepal. Facilitators received training during April 2024 on the ALIVE intervention concepts, foundational helping skills, basic facilitation skills, role-plays for selected intervention sessions, adverse events (such as those at risk of suicidality) identification, reporting, and management, and ways to overcome social stigma attached to mental health issues by using ‘heart-mind’ terminologies (such as “*man* [heart-mind] instead of *dimaag* [brain-mind]”, “*manosamajik samasya* [psychosocial problems] instead of *maanasik rog* [mental illness]”). Additionally, facilitators in the self-regulation and combined intervention arms received 14 h of training in martial-arts-based physical activities (which are part of both of these interventions), delivered in two-hour sessions over seven days. Finally, dyads of trained facilitators were assigned to schools for intervention implementation. The facilitators were young adults aged between 24 and 27 years, and fluent in Nepali. For the economic intervention arm, facilitators were required to have at least an intermediate degree or a bachelor’s degree in arts such as business studies or commerce. For the self-regulation and combined arms, facilitators were required to have an intermediate/bachelor’s degree in nursing or a bachelor’s degree in public health/social work or a related field (not limited to psychology or psychiatry). Prior experience with psychosocial or economic interventions was not required. During the implementation of the three active interventions, in-person supervision sessions with facilitators were organized by the intervention trainers. Weekly supervision sessions were organized to review the previous session, address any challenges encountered during intervention delivery, and prepare for the next session, including practicing key activities through role-plays.

#### Self-regulation intervention

The self-regulation intervention combined group physical activity with core active components. The purpose of this intervention was to strengthen self-regulation, i.e. enhancing the ability to set goals, manage the emotional impact of failure, and adjust behavior to achieve those goals, among adolescents by: (i) providing access to caring adults (facilitators) and a supportive peer group; (ii) building a positive self-concept by independently mastering challenging new tasks, including martial arts-based physical activity, and; (iii) offering relief from the stress caused by the adversity they experience daily. The intervention included four phases: phase 1 as caring facilitator behaviors, phase 2 as building strong connections, phase 3 as building strong minds, and phase 4 as building a strong future.

#### Economic intervention

The economic intervention consisted of cash plus intervention and targeted specific financial behaviors related to saving, budgeting, expense tracking, negotiation, and investment in education, among adolescents in three phases. In the first phase, the intervention focused on strengthening practical financial management skills, including budgeting, saving, and tracking expenses, which were tailored to the day-to-day financial needs and wants of adolescents. These behaviors were emphasized to promote both short-term and long-term financial planning. The second phase addressed negotiation skills, aiming to improve behaviors related to bargaining, identifying opportunities, and collaboratively seeking solutions. In the third phase, the intervention encouraged informed decision-making regarding investments in education by providing information on the returns to education, potential challenges, and strategies to overcome them. As part of the cash transfer component of the intervention, adolescents received small cash transfers, equivalent to NPR 2000 (i.e., 1/3 of the cash amount, approximately $15), six times over the intervention period, with the expectation that the combination of financial education and cash support would encourage responsible behaviors in money management and decision-making. The caregivers of the adolescents received the remaining 2/3 of the cash amount. Based on existing literature [83–88], the cash transfer amount was set equivalent to approximately 8% of per capita expenditure (PCE), which aligns with the average household transfer observed in similar programs, and distributed evenly over the six-month intervention period. Following Baird and colleagues [89], the transfer amount was split between the adolescent and parents in a 1:2 ratio, with one-third allocated to the adolescent and two-thirds to the parents. Cash transfers have increasingly been considered a “minimum floor” to provide transient poverty relief and create the conditions for households and adolescents to benefit from additional intervention components [90]– [91]. The cash transfer and financial training module of the economic intervention was designed to address income and budgeting constraints, while its integration with negotiation skills and information on returns to education was expected to produce a broader impact on adolescent mental health by addressing multiple pathways through which poverty contributes to depression and anxiety.

#### Combined intervention

The combined (economic + self-regulation) intervention included all core elements of the other two interventions but presented in a condensed format to fit the 120-minute intended duration. This combined intervention allowed for a broader learning scope for the adolescents, including emotional regulation, problem-solving, goal-setting skills, as well as cash transfer, financial literacy, negotiation and conflict resolution skills, and understanding the importance of education. Furthermore, the intervention included a section of approximately 5 min to explore the connections between the economic and self-regulation contents of the intervention. A detailed manual of the interventions can be found on the ALIVE webpage (https://www.alive4mentalhealth.org/manuals).

### Data analysis

Means, standard deviation (SD), proportions, and bivariate associations were examined for the socio-demographic characteristics at baseline per study arm to check whether randomization resulted in an even distribution across the arms. Bivariate correlations were performed between the key measures at baseline. We used SPSS (version 29.0) [92] and the PROCESS macro (version 4.2) to conduct data analysis and examine mediation effects in the parallel mediation model, separately for depression and anxiety outcomes as two separate models. To assess changes in resilience (for both internal and resilience measures) within-group differences across timepoints (T0, T1, T2), we examined linear mixed models (LMM) with individual-level random effects to account for correlation between repeated measures. We also explored whether resilience as an outcome changed within-group across time-points by gender. As an exploratory analysis, pairwise comparisons were also performed to identify which groups differ significantly at various time points. For mediation analysis, dummy variables were used for the predictor (intervention arms with reference as control, and D1, D2, D3 being self-regulation, economic, and combined arm, respectively). External resilience (CYRM-R total score at T1) and internal resilience (RRM score at T1) were treated as mediators simultaneously in the parallel mediation models, after confirming that there were no serious multicollinearity issues between the two mediators by examining the variance inflation factor (VIF) and tolerance. Depressive symptoms (PHQ-A score at T2) and anxiety symptoms (GAD-7 score at T2) were treated as two separate outcomes in the two parallel mediation models. Gender, baseline (T0) resilience measures, and baseline (T0) outcome measures were treated as covariates in the parallel mediation models. The mediation analysis involved the study of unstandardized coefficients, standard errors, and lower and upper limits. The PROCESS macro employed bootstrap calculations to provide bias-corrected 95% confidence intervals for the indirect effects, based on 5,000 samples. Statistical significance was established when the estimated 95% bootstrap confidence intervals did not include zero. Based on the modern mediation perspective, indirect effects were explored irrespective of the direct effect of interventions on outcome measures [49, 93–95]. The statistical analysis plan was pre-registered before analysis on Open Science Framework (OSF) (10.17605/OSF.IO/P5ABN).

### Handling missing data

Attrition at T2 was relatively low (18/229, 7.8%). We examined whether dropouts differed from those who completed the T2 assessment on age, gender, and baseline mental health outcomes using independent samples t-tests, χ²-tests, and logistic regression. No significant differences were found for age, gender, and baseline mental health outcomes (*p* > 0.05). Longitudinal analyses of resilience used linear mixed models with maximum likelihood estimation, which incorporate all available data under the assumption of missing at random. Mediation analyses were conducted using Hayes’ PROCESS macro (Model 4), which uses listwise deletion. Comparisons of included versus excluded participants suggested no systematic differences in baseline variables. Moreover, gender, baseline resilience, and outcome variables were controlled in the mediation analysis.

### Participant safety and clinical referrals

Any participants identified with depression (PHQ-A score ≥ 15), anxiety (GAD-7 score ≥ 10), or at risk of suicidality according to the ALIVE adverse event reporting and management (AERM) protocol were referred to a psychosocial counsellor for further clinical assessment and management. High-risk cases, as determined through clinical assessment by the counsellor, were provided with appropriate mental health and psychosocial support (MHPSS) services. At the first follow-up (T1), no participants required referral; however, at the second follow-up (T2), six participants were identified as being at risk of suicidality (one from the economic arm, two from the combined arm, and three from the control arm) and were referred for further care from the counsellor and, where necessary, a psychologist/psychiatrist. Additionally, there was one death in the control arm due to a road traffic accident, unrelated to the ALIVE study; in this case, the counsellor reached out to the parents to provide grief and bereavement support. Although intervention facilitators were trained to identify adverse events such as suicidality, depression, or anxiety during the intervention, no such cases were detected throughout the intervention period. The reports related to the serious adverse events were sent to the ALIVE Data Safety Management Board (DSMB), independent of the ALIVE research team, consisting of an independent statistician from the United Kingdom, three mental health experts, one each from Nepal, South Africa, and Colombia, who ascertained whether the event was a result of participation in the ALIVE trial, and whether the adequate response was taken.

## Results

### Baseline characteristics of the study participants

Table 1 shows sample characteristics across the four arms of the study and depicts that there was no difference in baseline demographics, outcomes, and mediator, except for the baseline external resilience measure. The paired comparison indicated a significant difference in baseline external resilience between the control and combined groups (*p* = 0.018).

**Table 1:** Adolescents’ baseline characteristics across each intervention arm and the control arm

	Total (*n* = 229)	Control (*n* = 60)	Self-regulation (*n* = 51)	Economic (*n* = 59)	Combined (*n* = 59)	*p*-value
	Mean (SD) or Frequency (%)	Mean (SD) or Frequency (%)	Mean (SD) or Frequency (%)	Mean (SD) or Frequency (%)	Mean (SD) or Frequency (%)	
Age (In years)	13.9 (0.73)	13.78 (0.72)	14.06 (0.79)	14 (0.64)	13.8 (0.76)	0.101^a^
Sex						
Male	113 (49.3)	27 (45)	24 (47.1)	34 (57.6)	28 (47.5)	0.520^b^
Female	116 (50.7)	33 (50)	27 (52.9)	25 (42.4)	31 (52.5)	
Grade						
Sixth/Seventh	44 (19.2)	14 (23.3)	9 (17.6)	8 (13.6)	13 (22)	0.313^b^
Eighth	103 (45)	21 (35)	26 (51)	33 (55.9)	23 (39)	
Ninth	82 (35.8)	25 (41.7)	16 (31.4)	18 (30.5)	23 (39)	
Poverty score (selection phase 1)	0.59 (0.14)	0.59 (0.14)	0.59 (0.14)	0.61 (0.16)	0.59 (0.13)	0.877^a^
Depressive symptoms	4.90 (2.77)	4.52 (2.86)	5.12 (2.78)	5.22 (2.78)	4.8 (2.68)	0.509^a^
Anxiety symptoms	3.16 (2.16)	2.98 (2.18)	3.14 (2.12)	3.39 (2.51)	3.14 (1.81)	0.784^a^
Internal resilience	37.77 (5.53)	38.65 (5.55)	37.57 (5.22)	38.27 (5.78)	36.54 (5.41)	0.173^a^
External resilience	68.15 (7.62)	69.82 (7.66)	68.61 (7.38)	68.54 (7.52)	65.68 (7.48)	0.023*^a^

^a^ One-way ANOVA, ^b^ Chi-square test, * significant at 0.05 level

### Bivariate correlations between measures at baseline

The findings in Table 2 show that there was a small but positive correlation between gender and depressive symptoms (*r* = 0.16, *p* = 0.019) and anxiety symptoms (*r* = 0.14, *p* = 0.036), suggesting that females reported higher depressive and anxiety symptoms than males at baseline. There was a marginal negative correlation between gender and internal resilience (*r*=-0.13, *p* = 0.047), indicating that females reported lower internal resilience than males. Difficulty in self-regulation had a moderately positive correlation with both depressive and anxiety symptoms (*r* = 0.38, *p* < 0.001), indicating higher the difficulties in self-regulation, the higher the symptoms. Similarly, difficulty in self-regulation was negatively correlated with internal resilience (*r*=-0.18, *p* = 0.006). Internal and external resilience were negatively correlated with both depressive and anxiety symptoms.

**Table 2:** Correlations between measures at baseline

	Gender	T0 PHQA	T0 GAD7	T0 DERS	T0 CYRMR	T0 RRM
Gender	–					
T0 PHQA	0.16*	-				
T0 GAD7	0.14*	0.63**	–			
T0 DERS	0.10	0.38**	0.38**	-		
T0 CYRMR	0.02	– 0.25**	– 0.19**	– 0.10	–	
T0 RRM	– 0.13*	– 0.28**	– 0.19*	– 0.18**	0.68**	–

*Correlation significant at 0.05 level, ** correlation significant at 0.01 level

### Sensitivity to change for external resilience and internal resilience as exploratory outcomes

The mean, standard error (SE), 95% confidence interval, mean difference (MD), and Cohen’s *d* for external resilience and internal resilience across the four study arms by timepoints (T0, T1, and T2) and gender are presented in Tables 3 and 4. The findings of the linear mixed model for external resilience as an exploratory outcome showed the overall fixed effects as containing (a) no significant main effect of time (*p* = 0.420), indicating that external resilience scores did not change substantially across the measured timepoints; (b) no significant interactions involving time (i.e., arm × timepoint [*p* = 0.469], timepoint × gender [*p* = 0.936], or arm × timepoint × gender [*p* = 0.653]), suggesting that resilience trajectories over time were similar across arms and genders, and (c) a significant arm × gender interaction (*p* = 0.046) suggests that the effect of group on external resilience varied by gender, while looking at the whole sample. Overall, there were no significant changes (mean deviation) in the hypothesized direction on external resilience as an outcome in all four groups (p-values > 0.05). However, exploratory post-hoc comparisons indicated non-significant improvements in external resilience over time, aligning with the hypothesized direction of the intervention, particularly in the economic arm for females (both T0 to T1 and T0 to T2) and males (T0 to T1 only), and in the self-regulation arm for males (T0 to T2).

**Table 3:** Estimated marginal means, SE, and mean difference in external resilience within-arms, by time-points, and by gender

Arm	Timepoint	Gender	External Resilience		Pairwise comparison (T1-T0 or T2-T0)		Effect size
			Mean (SE)	95% CI	MD (SE)	*p*-value	Cohen’s *d*
Control	T0	Male	70.89 (1.47)	67.99–73.78			
		Female	68.94 (1.33)	66.32–71.56			
		All	69.82 (0.99)	67.86–71.77			
	T1	Male	70.44 (1.47)	67.55–73.34		0.706	– 0.07
		Female	68.29 (1.33)	65.68–70.91	– 0.65 (1.07)	0.545	– 0.11
		All	69.26 (0.99)	67.31–71.21	– 0.56 (0.79)	-0.44 (1.18) 0.480	– 0.09
	T2	Male	70.85 (1.52)	67.87–73.84	– 0.04 (1.41)	0.979	– 0.01
		Female	67.44 (1.36)	64.76–70.12	– 1.50 (1.26)	0.235	– 0.22
		All	68.96 (1.02)	66.96–70.97	– 0.86 (0.94)	0.361	– 0.12
Self-regulation	T0	Male	69.50 (1.56)	66.43–72.57			
		Female	67.82 (1.47)	64.92–70.71			
		All	68.61 (1.08)	66.49–70.73			
	T1	Male	69.47 (1.58)	66.36–72.57	– 0.03 (1.27)	0.979	– 0.01
		Female	67.30 (1.49)	64.38–70.22	– 0.52 (1.20)	0.667	– 0.09
		All	68.32 (1.09)	66.18–70.46	– 0.29 (0.87)	0.740	– 0.05
	T2	Male	70.02 (1.60)	66.87–73.17	0.52 (1.48)	0.726	0.08
		Female	66.71 (1.51)	63.75–69.67	– 1.11 (1.39)	0.427	– 0.16
		All	68.26 (1.10)	66.09–70.43	– .35 (1.01)	0.731	– 0.05
Economic	T0	Male	67.91 (1.31)	65.33–70.49			
		Female	69.40 (1.53)	66.39–72.41			
		All	68.54 (1.00)	66.57–70.51			
	T1	Male	69.50 (1.31)	66.92–72.08	1.59 (1.05)	0.131	0.26
		Female	70.04 (1.53)	67.03–73.05	0.64 (1.22)	0.601	0.10
		All	69.73 (1.00)	67.76–71.70	1.19 (0.79)	0.135	0.20
	T2	Male	66.58 (1.32)	63.98–69.18	– 1.33 (1.22)	0.275	– 0.19
		Female	69.71 (1.56)	66.64–72.79	0.31 (1.45)	0.828	0.04
		All	67.87 (1.02)	65.88–69.87	– 0.67 (0.93)	0.472	– 0.10
Combined	T0	Male	64.11 (1.44)	61.27–66.95			
		Female	67.10 (1.37)	64.40–69.80			
		All	65.68 (1.00)	63.71–67.65			
	T1	Male	62.53 (1.46)	59.66–65.40	– 1.58 (1.17)	0.179	– 0.26
		Female	66.36 (1.37)	63.66–69.05	– 0.74 (1.10)	0.500	– 0.12
		All	64.55 (1.01)	62.58–66.53	– 1.12 (0.80)	0.160	– 0.18
	T2	Male	63.15 (1.49)	60.22–66.09	– 0.95 (1.38)	0.491	– 0.14
		Female	66.20 (1.40)	63.46–68.95	– 0.89 (1.29)	0.488	– 0.13
		All	64.77 (1.01)	62.75–66.78	– 0.91 (0.94)	0.332	– 0.13

The linear mixed model for internal resilience as an exploratory outcome showed the following overall fixed effects: (a) significant main effect of time (*p* = 0.027), indicating that internal resilience scores changed across the measured timepoints for the whole sample, suggesting the study period had an overall effect; (b) gender differences in internal resilience scores across the total sample (*p* = 0.007); (c) no significant interactions involving time (i.e., arm × timepoint [*p* = 0.107], timepoint × gender [*p* = 0.830], or arm × timepoint × gender [*p* = 0.261]), or arm × gender (*p* = 0.932) suggesting that resilience trajectories over time were similar across arms and genders, when looking at the whole sample. Overall mean deviation, in contrast to our hypothesized direction, showed that internal resilience reduced by 1.76 points irrespective of gender (*p* = 0.013), and particularly internal resilience decreased by 2.57 points (*p* = 0.020) among females in the economic intervention arm from T0 to T2; and in the combined arm, the internal resilience reduced by 2.01 points (*p* = 0.018) among males from T0 to T1, but this reduction was not statistically significant at T2. However, the estimated marginal means showed non-significant improvements in internal resilience over time, aligning with the hypothesized direction of intervention, particularly in the economic arm for males from T0 to T1, from T1 to T2 for males in the combined arm, and both males and females in the self-regulation arm.

**Table 4:** Estimated marginal means, SE, and mean difference in internal resilience within-arms, by time-points, and by gender

Arm	Timepoint	Gender	Internal Resilience		Pairwise comparison (T1-T0 or T2-T0)		Effect size
			Mean (SE)	95% CI	MD (SE)	*p*-value	Cohen’s *d*
Control	T0	Male	39.37 (1.04)	37.33–41.41			
		Female	38.06 (0.94)	36.21–39.91			
		All	38.65 (0.70)	37.27–40.03			
	T1	Male	39.56 (1.04)	37.51–41.60	0.19 (0.85)	0.828	0.04
		Female	37.03 (0.94)	35.18–38.88	– 1.03 (0.77)	0.181	– 0.23
		All	38.17 (0.70)	36.79–39.54	– 0.48 (0.57)	0.398	– 0.11
	T2	Male	38.24 (1.07)	36.13–40.35	– 1.13 (1.07)	0.293	– 0.22
		Female	36.70 (0.97)	34.81–38.60	– 1.36 (0.96)	0.159	– 0.26
		All	37.39 (0.72)	35.97–38.81	– 1.26 (0.71)	0.078	– 0.24
Self-regulation	T0	Male	38.88 (1.10)	36.71–41.04			
		Female	36.41 (1.04)	34.37–38.45			
		All	37.57 (0.76)	36.08–39.06			
	T1	Male	38.25 (1.12)	36.06–40.44	– 0.63 (0.92)	0.496	– 0.14
		Female	36.08 (1.05)	34.02–38.14	– 0.33 (0.86)	0.705	– 0.08
		All	37.10 (0.77)	35.59–38.61	– 0.47 (0.63)	0.458	– 0.11
	T2	Male	38.45 (1.13)	36.23–40.68	– 0.42 (1.13)	0.710	– 0.08
		Female	36.28 (1.07)	34.19–38.38	– 0.13 (1.06)	0.905	– 0.02
		All	37.30 (0.78)	35.77–38.83	– 0.27 (0.77)	0.726	– 0.05
Economic	T0	Male	38.50 (0.93)	36.68–40.32			
		Female	37.96 (1.08)	35.84–40.08			
		All	38.27 (0.71)	36.88–39.66			
	T1	Male	39.32 (0.93)	37.50-41.14	0.82 (0.76)	0.278	0.19
		Female	37.16 (1.08)	35.04–39.28	– 0.80 (0.88)	0.366	– 0.18
		All	38.41 (0.71)	37.02–39.80	0.14 (0.58)	0.814	0.03
	T2	Male	37.32 (0.93)	35.48–39.15	– 1.19 (0.93)	0.204	– 0.22
		Female	35.39 (1.11)	33.22–37.56	– 2.57 (1.10)	0.020*	– 0.49
		All	36.51 (0.72)	35.10-37.92	– 1.76 (0.71)	0.013*	– 0.33
Combined	T0	Male	37.36 (1.02)	35.35–39.36			
		Female	35.81 (0.97)	33.90-37.71			
		All	36.54 (0.71)	35.15–37.93			
	T1	Male	35.35 (1.03)	33.32–37.38	– 2.01 (0.85)	0.018*	– 0.45
		Female	35.58 (0.97)	33.68–37.49	– 0.23 (0.79)	0.776	– 0.05
		All	35.48 (0.71)	34.09–36.88	– 1.06 (0.58)	0.069	– 0.24
	T2	Male	37.02 (1.06)	34.94–39.10	– 0.34 (1.05)	0.748	– 0.06
		Female	35.01 (0.99)	33.07–36.95	– 0.80 (0.98)	0.417	– 0.15
		All	35.94 (0.73)	34.52–37.37	– 0.60 (0.72)	0.404	– 0.11

*Significant at 0.05 level

### Exploration of mediational effects of internal and external resilience between study arms and depression and anxiety symptoms

**Fig. 2:**
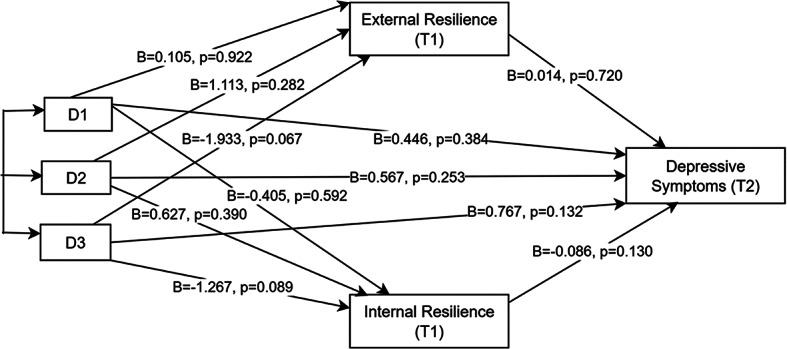
Path diagram showing the mediation effect of resilience (T1) on the relationship between the intervention arm and depressive symptoms at T2. D1, D2, D3 refer to dummy coded variables for self-regulation, economic, and combined arm, respectively, in reference to the control group

**Fig. 3:**
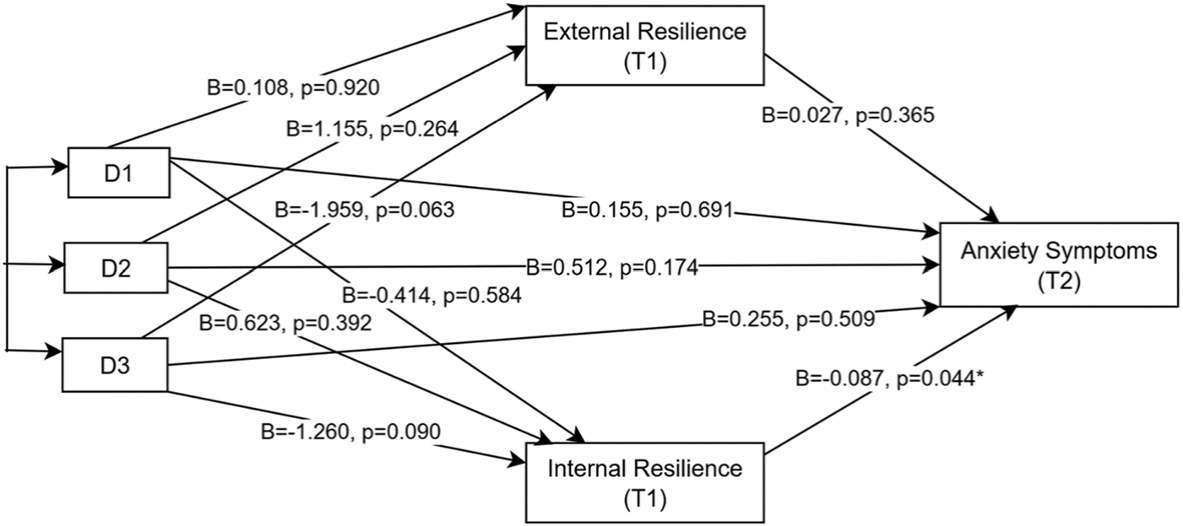
Path diagram showing the mediation effect of resilience (T1) on the relationship between the intervention arm and anxiety symptoms at T2. *Significant at 0.05 level; D1, D2, D3 refer to dummy coded variables for self-regulation, economic, and combined arm, respectively, in reference to the control group

Internal and external resilience at T1 were positively correlated (*r* = 0.66); however, the variance inflation factor (VIF = 1.7) and tolerance (0.56) between them confirmed that multicollinearity issues were absent and were therefore appropriate to treat them as separate mediators in the parallel mediation models. The results of the exploratory mediation analyses (Figs. 2 and 3) using indicator coding with reference as the control arm indicated that there was no significant association between intervention arm and depressive and anxiety symptoms at T2, neither directly nor through internal or external resilience in any of the intervention arms. There was a small, significant effect of internal resilience (T1) on T2 anxiety symptoms in all three intervention arms with reference to the control group (i.e., b– path unstandardized coefficient =– 0.087, *p* = 0.044). For both depressive and anxiety symptoms at T2, gender included as a covariate in the direct effect model showed significant effects: B = 0.907, *p* = 0.016 for depression, and B = 0.894, *p* = 0.002 for anxiety.

When further exploration by subgroup analysis per gender was conducted, the mediation results showed that, for females, there were no effects, neither direct nor through resilience, for T2 depressive and anxiety symptoms in all intervention arms relative to the control arm. For males, some notable effects emerged, particularly for depression as the outcome. In the self-regulation and economic arms, the b-path (from internal resilience to depression) was significant (B = – 0.156, *p* = 0.049), indicating that higher internal resilience was associated with lower depressive symptoms. However, other paths in these arms (a-paths and indirect effects) were not significant. In the combined arm, several paths were noteworthy. The a-paths (from intervention to resilience) were significant for both types of resilience: external resilience (B = – 3.640, *p* = 0.023) and internal resilience (B = – 2.367, *p* = 0.042), indicating unexpected, reduced resilience at T1 for males in the combined arm as compared to the control group. The b-path for internal resilience remained significant (B = – 0.156, *p* = 0.049), linking higher internal resilience to lower depression. However, the b-path for external resilience and the indirect (ab) effects—whether via internal or external resilience—were not significant. The direct effect (c-path) was not significant, but the total effect (c′-path) was significant (B = 1.422, *p* = 0.044), suggesting a significant overall effect of the combined intervention on depressive symptoms, even though the mediation paths did not fully explain this effect.

## Discussion

Setting and pursuing goals—whether economic or emotional—despite challenges, was the key focus of the ALIVE interventions—self-regulation, economic, or combined—to strengthen motivation and goal-setting abilities [21], which are important resilience factors. Accordingly, this study explored (i) sensitivity to change in resilience from T0 to T1 and T0 to T2, (ii) the potential mediating role of resilience at 6 months (T1) between ALIVE interventions and mental health outcomes at 12 months (T2), and (iii) gender differences in these exploratory effects.

Although there were no significant changes in the hypothesized direction on external resilience as an outcome within-intervention groups, a possible positive change direction was observed for males in the self-regulation (baseline to second follow-up) and the economic arm (baseline to first follow-up), and females in the self-regulation arm (baseline to both follow-ups), suggesting potential time-related positive changes in external resilience. Despite significant decreases in internal resilience among females in the economic intervention arm (from baseline to second follow-up) and among males in the combined arm (from baseline to first follow-up), we observed a possible positive change in direction. These included positive improvements, though not significant, in internal resilience for males in the economic arm from baseline to first follow-up, for males in the combined arm from first to second follow-up, and for both males and females in the self-regulation arm from first to second follow-up, indicating potential time-related gains in internal resilience that warrant further investigations. The exploratory mediation analyses revealed that there was no significant direct or indirect effect of the interventions via resilience on depressive and anxiety symptoms at 12-month follow-up, though internal resilience at 6 months had a small, significant effect on hypothesized direction (i.e., b-path) on anxiety symptoms at 12 months across all intervention arms compared to the control arm. The gender-wise analysis showed that in contrast to the hypothesized direction, for males in the combined arm compared to the control group, the direct pathway from intervention to resilience was negatively correlated; however, in all three intervention arms (self-regulation, economic, and combined) for males, the direct-pathway from internal resilience toward depression was significantly negatively associated, as hypothesized, indicative of higher the internal resilience lower would be the depression.

These results suggest that, within the scope and time frame of this study, the intervention did not significantly measurably change resilience, nor did resilience act as a key explanatory pathway for the observed mental health outcomes. However, while the observed changes were not statistically significant, the direction of effect supports the theoretical pathway underpinning the ALIVE intervention, leading to a possible change in resilience, particularly internal resilience first as an outcome and then as a process correlated with reduced anxiety symptoms for both males and females and reduced depression for males. As noted, this pilot study was intentionally underpowered and not designed to assess the fit of our data with the conceptually testable mediation models depicted in Figs. 2 and 3. Because our model aligns closely with core assumptions of prominent resilience theories—including the ecological resilience model [63–66], the developmental systems framework [75], and the adaptation-based approach [76]—the current study demonstrates its conceptual robustness. By extending the model beyond its original focus on ecological resources, including both internal and external protective factors of individual resilience, and by incorporating cash transfers and other economic components (in the economic and combined interventions), our study highlights the conceptual model’s utility as a testable, interdisciplinary framework for understanding resilience processes and mechanisms in preventive interventions for adolescents in LMIC contexts such as Nepal. In doing so, this study contributes to intervention science by illustrating how theoretically grounded, contextually adapted models can inform the design and evaluation of mental health prevention strategies for adolescents in resource-limited settings. In this study, the participants showed potential time-related positive changes in direction, in resilience. This finding aligns with prior research demonstrating that interventions targeting protective factors such as cognitive competence, problem solving/ decision making, cooperation and communication, and coping skills within cognitive behavioral therapy approach can significantly strengthen resilience among adolescents [55, 96]. A previous study involving selective transdiagnostic prevention interventions found slight improvement in resilience after 6-month post-intervention follow-up, which increased to a larger effect size after the booster session [97]. Prior studies have also shown that cash transfers resulted in improved resilience compared to a control group [98], improved perceptions of positive future outlook (optimism) [99], and a stress management intervention among Greek adolescents, which showed significant outcomes through improved resilience compared to the control group [100]. However, unlike previous studies that focused explicitly on building resilience [55, 96, 98, 100], the current study—being a pilot and underpowered trial—not only did not show significant effects but also did not aim to measure effectiveness.

Moreover, while the ALIVE intervention included components such as emotion regulation, negotiation, financial literacy, and educational information—conceptually linked to protective factors of resilience—it did not explicitly target resilience as a primary outcome. This indirect approach may have resulted in insufficient or diffuse effects on resilience, leading to no measurable change in effect size in our study from baseline. The second factor to consider is that resilience is shaped by cumulative and long-term influences from factors such as family, community, and systemic support structures, including institutional support from school, peer support [57, 62, 64–66], and culture and norms encouraging help-seeking and reducing stigma attached to mental health issues, particularly in the context of low-resource settings such as Nepal. The immediate post-intervention (T1) and 12-month follow-up (post-baseline) (T2) periods may not have been sufficient to capture meaningful shifts in resilience, especially from interventions that only indirectly touch on protective factors, as seen in other studies that show delayed rather than immediate resilience gains [101]. Third, the selective prevention nature of the intervention may have also affected external protective factors like peer support and stronger social connectedness, as various qualitative studies highlighted that adolescents undergoing selective school-based mental health interventions frequently report anticipated and experienced stigma, social isolation by creating a divide between those who receive interventions and those who don’t [102]. Nevertheless, in the current study, the self-regulation and combined interventions were designed to reduce isolation and thereby prevent depression/anxiety and included substantial components focused on building psychologically safe spaces in groups, building trust, teaching communication skills, and role modelling caring behaviours by the facilitators. Future studies should consider these factors when designing preventive interventions for adolescents, as well as for the economic intervention arms. Moreover, the research assistants conducting the interviews for data collection and intervention facilitators implementing the interventions were trained on ways to minimise social stigma associated with mental ill health by using socio-culturally accepted terminologies related to psychosocial health, such as the heart-mind concept, as suggested by existing evidence in the context of Nepal [103].

The absence of significant mediation effects of resilience in the exploratory mediation analyses in the present study indicate that there may be other pathways of change which could be responsible in the ALIVE intervention that were not captured by the resilience measures used- such as behavioral activation [104, 105] and cognitive reappraisal- a specific type of cognitive reframing strategy [106] in reducing depression. Other possible mechanisms to be explored include reduced emotional dysregulation, increased hope, reduced parental distress, improved parenting, or enhanced family communication that may have contributed more directly to changes in adolescent depression and anxiety outcomes. The findings of the present study also suggest that the external resilience, which depends on external support systems, may not improve immediately after an intervention and could require a longer time to develop. In contrast, internal resilience, built on protective factors such as problem-solving abilities, emotion regulation, and coping skills, may show earlier improvements due to the intervention, which in turn could help reduce anxiety symptoms at 12 months.

Considering the recommendations from the previous studies to report the effects of gender in adolescent mental health intervention [15], we conducted subgroup analysis by gender. For females, no intervention effects were observed. For males, particularly in the combined intervention arm, improvements in internal resilience at post-intervention (T1) were significantly associated with reduced depressive symptoms at T2. However, mediation pathways were only partially supported, and the overall effect remained significant despite non-significant indirect paths. The difference in direction and effects observed between male and female adolescents may be influenced by contextual and cultural factors in Nepal. Traditional masculine norms often discourage emotional expression and self-reflection among males; thus, exposure to both economic and self-regulation interventions may have contributed to a modest short-term improvement in their internal resilience. In contrast, external resilience likely requires more time to develop, as it depends on sustained support from the surrounding environment. Additionally, persistent gender equity concerns in Nepal may disadvantage females, potentially limiting the development of resilience processes in this group. As indicated in our previous study among Nepalese adolescents from the ALIVE formative research, the stressors and coping strategies were shaped by contextual factors across socio-ecological levels such as peer relationships, school dynamics, or family stressors, gender differences in coping leading to different resilience processes in boys and girls, such as girls leaning more towards avoidance-escape and helplessness, whereas boys favoring opposition and isolation [107]. This may have influenced the outcomes in the current study and contributed to the lack of observable changes in resilience. However, as indicated by trends in direction in the exploratory findings of this study, the potential role of resilience—whether internal or external—as a mechanism of change needs to be confirmed in a future fully powered definitive ALIVE trial, alongside the examination of other possible pathways.

The preliminary findings suggest that the ALIVE intervention appears to have potential for scale-up across diverse settings in Nepal. However, the results from the feasibility evaluation of the ALIVE intervention will be reported separately. This paper focuses on resilience as a mediator and outcome of the pilot trial (rather than conducting a comprehensive evaluation of the intervention). Questions regarding scalability, including effectiveness, fidelity, and sustainability in real-world conditions, can only be comprehensively addressed in a future, larger hybrid implementation trial.

The findings of the current study highlight the need for further research that explicitly examines resilience processes in relation to social determinants of mental health, including how these structural factors interact with psychological processes to influence mental health outcomes. This study also highlights the importance of directly targeting resilience in intervention design, accounting for ecological and contextual influences, and incorporating longer follow-up periods. It is important to assess the long-term impact (at least 12-month follow-ups post-intervention) of mental health interventions to determine their sustainability over time [108]. A recent systematic review targeting the effects of selective prevention interventions on depression and anxiety among adolescents reported that moderate improvements occurred only at medium to long-term follow-ups [109]. A future ALIVE trial with a larger sample size and with appropriately powered statistical analysis should confirm the conceptual mediation model proposed by this study, with resilience as a process and mediator of the interventions. Future studies should also explore alternative mediators and mechanisms of change while addressing potential stigma effects and social dynamics in selective interventions. Similarly, the use of tailored resilience measures aligned with the intervention content and adapted to the cultural and contextual relevance is recommended for future studies.

There are several limitations in the present study that need to be accounted for while interpreting the findings. First, a key limitation of this study is its generalizability. The sample was relatively small and recruited exclusively from Kathmandu, which may not represent adolescents living in rural poverty in Nepal. Given the marked rural–urban disparities in access to resources, educational opportunities, and mental health awareness, adolescents in the Kathmandu valley may differ in ways that influence intervention responsiveness. Consequently, the findings should be interpreted with caution when considering broader application across diverse geographic and socio-economic contexts in Nepal. Second, the relatively sample size led to analyses that were underpowered. Third, there were only 3-time points involved (baseline, 6-month post-baseline, and 12-month post-baseline), while the sustained effects of the mental health intervention may appear at a later stage. Fourth, self-report data are subject to biases and may have led to social desirability response bias. Fifth, the baseline measure of external resilience differed across the study groups, particularly between the combined and control groups; however, as the baseline resilience score was controlled for in the exploratory mediation analyses, the results were unlikely to have been affected. Nevertheless, we suggest that in a future trial, the socio-ecological factors offered by the school-related things be examined and/or controlled. Sixth, the study did not assess adolescents’ prior mental health conditions or use of psychotropic medications. Pre-existing mental health problems or ongoing treatment could influence both baseline resilience and responsiveness to the intervention, and their absence from the data limits the ability to fully account for these potential confounding factors in the analyses. Lastly, the variation in the intervention session implementation timing across the arms may have influenced participants’ engagement or responsiveness, potentially affecting resilience and mental health outcomes (depression and anxiety symptoms). Future studies should standardize session timing or systematically examine its impact on intervention.

## Conclusion

Although there were no significant changes in external resilience from baseline to the second follow-up at 12 months, marginal positive trends over time were observed across some gender and intervention subgroups, particularly for males and females in the self-regulation arm. Internal resilience showed some significant declines in specific groups, but also indicated possible improvements in hypothesized direction over time in others, suggesting potential time-related gains in the ALIVE intervention deserving of further exploration. Mediation analyses revealed no significant indirect effects of interventions via external resilience on depression or anxiety; however, internal resilience at 6 months had a small but significant effect on anxiety at 12 months, and for males, internal resilience was significantly and negatively associated with depression across intervention arms, suggesting a testable mediation model which could be examined in future mental health selective prevention interventions for adolescents. Future definitive trials should replicate this proposed mediation model with resilience as a mechanism in a larger sample with powered analysis and for a longer follow-up period, of at least 12- or 18-month post-intervention.

## Data Availability

All required data are available within the manuscript. The data that support the findings of this study are part of a larger research project and are not publicly available for ethical reasons. Further details regarding the intervention contents can be found in the manual for the ALIVE project at (https://www.alive4mentalhealth.org/manuals).
